# Estimating causal effects of internet interventions in the context of nonadherence

**DOI:** 10.1016/j.invent.2020.100346

**Published:** 2020-08-29

**Authors:** Hugo Hesser

**Affiliations:** School of Law, Psychology and Social Work, Center for Health and Medical Psychology, Örebro University, Sweden; Department of Behavioural Sciences and Learning, Linköping University, Linköping, Sweden

**Keywords:** Complier average causal effect, Psychological treatment, Randomized trial, Mixture modeling, Adherence, Structural equation modeling

## Abstract

A substantial proportion of participants who are offered internet-based psychological treatments in randomized trials do not adhere and may therefore not receive treatment. Despite the availability of justified statistical methods for causal inference in such situations, researchers often rely on analytical strategies that either ignore adherence altogether or fail to provide causal estimands. The objective of this paper is to provide a gentle nontechnical introduction to complier average causal effect (CACE) analysis, which, under clear assumptions, can provide a causal estimate of the effect of treatment for a subsample of compliers. The article begins with a brief review of the potential outcome model for causal inference. After clarifying assumptions and model specifications for CACE in the latent variable framework, data from a previously published trial of an internet-based psychological treatment for irritable bowel syndrome are used to demonstrate CACE-analysis. Several model extensions are then briefly reviewed. The paper offers practical recommendations on how to analyze randomized trials of internet interventions in the context of nonadherence. It is argued that CACE-analysis, whenever it is considered appropriate, should be carried out as a complement to the standard intention-to-treat analysis and that the format of internet-based treatments is particularly well suited to such an analytical approach.

## Highlights

• Nonadherence is a common challenge in internet interventions.
• Inappropriate statistical methods for dealing with nonadherence are often applied.
• An overview of one appropriate method, complier average causal effect analysis.
• A latent variable framework makes the analytical approach very flexible.
• Practical recommendations for the analysis of randomized trials, including a motivating example.

## 1 Introduction

The randomized experiment is often the preferred method for evaluating the efficacy of a specific intervention. Over the last decades a substantial number of randomized controlled trials has been performed to evaluate psychological treatments delivered via the internet for a wide variety of health problems (Andersson et al., 2014, Andersson et al., 2019; Andrews et al., 2010). The format of intervention delivery has proven to be most useful with overall encouraging results in terms of efficacy and acceptability (see for review, Andersson, 2016). However, some participants, for one reason or another, do not adhere to the treatment that they are encouraged to take in internet interventions and may therefore not receive treatment. Nonadherence is an issue that has repeatedly been addressed in the field of internet interventions (e.g., Christensen et al., 2009; Donkin et al., 2011; Eysenbach, 2005; Kelders et al., 2012; Ryan et al., 2018). For example, an individual might drop out from a trial prior to being exposed to the core treatment components. What can be said about the treatment effect in such a scenario when the individual was never exposed to the treatment? The individual might have responded to treatment *had* he or she actually received treatment, but we simply do not know the outcome in this counterfactual scenario. Individuals who do not adhere to treatment are likely to differ on key characteristics from those who actually do adhere, making it a challenge to estimate the overall effect of treatment when some participants do not receive treatment. Thus, nonadherence to treatment constitutes a threat to the validity of causal claims in randomized experiments.

Adherence can refer to multiple things in a treatment trial. Adherence can be related to the overarching term *treatment implementation*, a concept that, in turn, is related to aspects such as whether the treatment was delivered as intended, whether the participant received the treatment and whether the participant actually did what he or she was expected to do in the treatment (Shadish et al., 2002). Often these aspects blend together in different definitions. This is also true for internet interventions where multiple definitions of adherence have been proposed (Kelders et al., 2012; Ryan et al., 2018). Importantly, adherence should not be confused with missing data, although they are often related concerns in a clinical trial (Christensen et al., 2009; Eysenbach, 2005; Jo et al., 2010). With adherence–here the term compliance is used interchangeably because that is the term most frequently used in the statistical literature–I refer to the process in which a participant adheres to the program in such a way that he or she *receives the treatment as intended*, regardless of whether outcome measures are completed. In certain words, in order to be identified as an adherer (or complier) in an internet intervention, a person should have been exposed to the core components or the content of the treatment, for example by reading certain text-based materials or interacting with certain web-components which, from the researcher's point of view, are considered essential for the intervention. This definition is similar to how others (e.g., Christensen et al., 2009; Eysenbach, 2005) have conceptualized adherence in internet interventions.

Nonadherence rates can be substantial in internet interventions (Christensen et al., 2009; Waller and Gilbody, 2009), but rates also vary widely across studies depending on therapist contact, format of intervention, type of nonadherence etc. (Kelders et al., 2012; van Ballegooijen et al., 2014). Although evidence available to date does not suggest that there are large differences in adherence rates between guided internet-based psychological treatments and face-to-face treatments (van Ballegooijen et al., 2014), it remains unclear whether format affects adherence due to few direct comparisons. What is clear, however, is at least to some degree, almost all clinical trials evaluating psychological treatments, regardless of the delivery format, suffer from nonadherence. Despite that measures of adherence (e.g., number of completed modules) have repeatedly been associated with outcomes following internet-based psychological treatments (Donkin et al., 2011), we still know little about how adherence rates can reliably be increased in internet interventions (Christensen et al., 2009). Thus, nonadherence is a common challenge in the analysis of trials of internet interventions, leaving researchers within the field with an unanswered question: How to best handle nonadherence by means of statistical methods? Although several previous attempts have been made to encourage the use of adequate statistical methods for handling missing data in internet interventions (e.g., Christensen et al., 2009; Hesser, 2015), little has been said about how to properly analyze clinical trials in which a subsample does not receive or take treatment in internet interventions. This is surprising because missing data and nonadherence represent two common problems that both need to be adequately addressed in randomized experiments in order to move from correlational conclusions into the realm of causal inference (Sagarin et al., 2014; Shadish et al., 2002).

Intention-to-treat (ITT) analysis is the standard way of analyzing data from randomized trials (Hollis and Campbell, 1999). ITT-analysis is based on all individuals who are randomized, regardless of the degree to which individuals comply with or adhere to the treatment. A less known fact among applied researchers is that ITT-analysis assumes that all individuals adhere to the treatment they are offered in order for it to provide an estimate of the causal effect of the treatment (Imbens and Rubin, 2015; Little and Yau, 1998). In trials with nonadherence, ITT-analysis provides, at best, an average unbiased effect of assignment (Imbens and Rubin, 2015; Little and Yau, 1998). Such an estimate might be interesting for policy decisions (Shadish et al., 2002), but it might also misrepresent the effect of the treatment itself. Thus, a substantive research question in clinical trials, in the presence of nonadherence, is what effect does treatment have on individuals who actually receive treatment (Imbens and Rubin, 2015; Jo et al., 2010; Little and Yau, 1998; Sagarin et al., 2014). ITT-analysis provides no such information if adherence is less than perfect and can subsequently misdirect further research efforts. In addition, if nonadherence occur together with missing data, the estimate obtained from ITT-analysis might also be biased (Frangakis and Rubin, 1999; Jo et al., 2010).

The challenge with adherence is that the variable is measured after assignment and may therefore jeopardize a fair comparison of conditions if inappropriately included in the analysis (Imbens and Rubin, 2015; Jo and Muthén, 2001; Sagarin et al., 2014). Commonly used subpar methods for dealing with nonadherence in clinical trials ignore the fact that adherence is a post-assignment variable that basically breaks randomization. For example, *as-treated* (which reassigns nonadherers to control) and *per-protocol* (which removes nonadherers from the analysis) are two widely used methods. Both approaches make the comparison unfair by pooling across groups of (potential) adherers and nonadherers in treatment and control (Jo, 2002a; Jo and Muthén, 2001) and generally do not provide causal estimates (Imbens and Rubin, 2015). In other words, these comparisons are problematic because (measured and unmeasured) variables are no longer balanced among the conditions, resulting in that outcomes for groups with potentially different characteristics are being compared. By simply conditioning on for example number of completed modules, as a proxy for treatment dosage, will in similar fashion produce biased estimates (c.f., Maracy and Dunn, 2011).

However, there are more sophisticated methods that can keep randomization intact, even if you condition on intermediate variables, such as for example adherence. There has been a growing interest in the development of such approaches, considering the practical and theoretical interest in studying the effects of treatment itself (relative to a control), not just the effect of assignment or of offering treatment (Hernán and Robins, 2017; Sagarin et al., 2014; Sobel and Muthén, 2012). In the following article, I will focus on one statistical method to handle nonadherence in randomized trials: complier average causal effect analysis (CACE; aka. local average causal treatment effect, LATE) (Angrist et al., 1996; Little and Yau, 1998). The method provides an unbiased estimate of the effect of assignment among those who actually receive treatment, that is, compliers or adherers. In other words, CACE-analysis compares individuals in the treatment condition who adhere to treatment with individuals in the control condition who *would have* adhered had they been assigned to treatment. Angrist et al. (1996) showed that, under certain assumptions, we can identify the causal effect and thus provide the effect of the treatment itself in this subsample of participants. Since then, numerous studies have successfully applied and expanded the analytical ideas to randomized trials of both medical and behavioral interventions in the statistical literature (Imbens and Rubin, 1997; Jo, 2002a; Little et al., 2009; Little and Yau, 1998; Stuart et al., 2008; Yau and Little, 2001) and, generally, these ideas have proved useful in the analysis of causal effects in the presence of nonadherence (VanderWeele, 2011). Thus, in order to get at more complete view of the effect of the treatment in randomized trials, CACE can, in certain situations, provide complimentary information.

Given that most researchers within the field are unaware of CACE-analysis and because it is underutilized in clinical trials examining internet interventions, I will provide a gentle, nontechnical introduction to CACE-analysis in this paper. Furthermore, I argue that internet interventions, which are generally highly structured treatments and are primarily based on text-based material with limited therapist involvement (Andersson, 2016), may be particularly well suited to CACE-analysis. That is, compared to face-to-face treatment it is easier to determine whether an individual has been exposed to the treatment content, as this is generally not dependent on the behavior of the therapist (Hesser et al., 2017). To do this in face-to-face psychological treatments integrity ratings of therapists delivering the treatment are required and in the vast majority of psychological treatment trials such ratings are missing (Perepletchikova et al., 2007), potentiality due to the methodological challenges involved. It could therefore be argued that the format of internet-based treatments simplifies the process of determining whether or not an individual has received treatment.

The paper is structured as follows. First, I will briefly present the potential outcome framework for causality that is of key importance for understanding CACE-analysis. Second, I will in nontechnical way present the basic idea behind CACE-analysis in the latent variable framework in terms of assumptions and model specifications. Third, I will go through an applied example of CACE-analysis using data from a previously published randomized trial of an internet-based treatment for irritable bowel syndrome. Finally, I will present some model extensions and end with a few recommendations for randomized trials evaluating internet interventions in the presence of nonadherence.

## 2 Causality within the potential outcome framework

Counterfactual reasoning is at the heart of causal inquires (Mackie, 1974; Morgan and Winship, 2007; Woodward, 2003). That is, the effect of a cause is the difference between what we did observe under certain conditions and what we *would have observed* under similar, but not identical conditions. The difference in the conditions is the cause. So, we need to conceive of a possible state of affairs where some action was not the same as that taken. Consider, for example, a situation in which John was treated with an internet-based treatment against depression and we could measure his level of depression after treatment. In order to view treatment as a potential cause of John's depression level, we also need to imagine a situation in which Johan *had not been* treated and his level of depression *would not have been* the same. More formally, if Z is a cause of Y, then if Z occurs then Y occurs, and if Z had not occurred then Y would not have occurred. The counterfactual is, by definition, unobserved and this makes causality a challenge. As Holland (1986) expressed it, this is “the fundamental problem of causal inference” (p. 947).

The potential outcome model (aka. Rubin's causal model; Holland, 1986; Imbens and Rubin, 2015; Rubin, 1974, Rubin, 2005) has formalized these general ideas of causality from a statistical point of view using formal and technical notations. As a broad, generic framework it provides details on the conditions under which we can expect to get unbiased estimates of causal effects. It also helps researchers to think clearly about causality and underlying assumptions of cause and effect associations in different situations. As such, the model has proven useful for dealing with post-assignment variables, such as for example noncompliance, in randomized experiments (Frangakis and Rubin, 2002). Despite these advances and decades of applied work in different areas, the potential outcome model is still not widespread among applied researchers, especially among those in the field of psychology. Before we turn to the issue of noncompliance let me go through the framework briefly. For a more in-depth treatment of the potential outcome model readers are referred to other sources (Imbens and Rubin, 2015; Morgan and Winship, 2007; Rubin, 2005).

Central concepts in the model are units, treatments and potential outcomes as well as assignment mechanisms (Imbens and Rubin, 2015). Consider a randomized trial, where treatment has two levels, treatment and control, and each individual, or unit, prior to being exposed to one of the conditions has a potential outcome under each condition at a particular point in time. Any unit exposed to treatment could have been exposed to control and vice versa. More formally this can be expressed with a treatment assignment status variable *Z*_*i*_, where *Z*_*i*_ = 1 if the individual *i* is randomly assigned treatment, and *Z*_*i*_ = 0 if assigned to control; likewise let *Y*_*i*_(1) denote the potential outcome for individual *i* when assigned to treatment and *Y*_*i*_(0) when assigned to control. The individual causal effect can then be defined as the difference in potential outcomes under these two conditions, *Y*_*i*_(1) − *Y*_*i*_(0). It is important to note that the causal effect is defined as a difference in two hypothetical outcomes under a treatment and control treatment and it is therefore relative to and dependent on the type of control. Once the individual has been exposed to one of the two treatments (treatment or control) only one of the two potential outcomes is realized (Holland, 1986; Imbens and Rubin, 2015). In essence, causal inference is viewed as a missing data problem, where the assignment mechanism, known or unknown by the researcher, determines which one of the two potential outcomes that is observed (Rubin, 1974). Subsequently, the causal effect can be defined at the individual level, but since the two outcomes can never be observed jointly for any unit, this causal effect can never be observed in practice (without assuming a constant treatment effect over multiple units) (Holland, 1986).

We can, however, learn about the causal effect using multiple units and by averaging over units that have been exposed to different treatment conditions (Imbens and Rubin, 2015; Rubin, 1974). The average causal effect (ACE) is the difference in potential outcomes between treatment and control, averaged over the entire population of units (Imbens and Rubin, 2015). Using expectations, we get the following general expression,

(1) $$\mathrm{ACE}=E\left[Y_{i}\left(1\right)-Y_{i}\left(0\right)\right].$$

Eq. (1) makes it clear that the potential outcome model is not restricted to any particular outcome, but for simplicity we can make use of a continuous outcome and a mean difference as the *causal estimand* of interest. Let *μ*_1_ denote the population potential mean outcome when assigned to the treatment condition (*Z* = 1), and *μ*_0_ when assigned to the control condition (*Z* = 0). The ITT effect, averaging over all units as randomized, can then be defined as,

(2) $$\mathrm{ITT}=\tau =\mu_{1}-\mu_{0}.$$

Once we have determined the ACE of primary interest, we can estimate the effect using a number of different analytic methods. For example, we can use a regression model to estimate *τ* for one continuous outcome measured post-treatment and a binary indicator variable for treatment assignment and any other pre-treatment variable that is related to the outcome (Imbens and Rubin, 2015) (e.g., pre-treatment value of the outcome, ANCOVA approach). A standard linear regression model is,

(3) $$Y_{i}=\beta_{0}+\beta_{1}Z_{i}+\beta_{2}X_{i}+e_{i},$$

where *Z*_*i*_ is a binary treatment indicator, *X*_*i*_ a covariate assessed at pretreatment, and *e*_*i*_ is a normally distributed residual. The treatment effect *β*_1_ is of primary interest in the model. Randomization of units to conditions ensures, in expectation, that the assignment mechanism is independent of the potential outcome of *Y*_*i*_ and the pretreatment chacteristics. In other words, units randomized to treatments will have identical distributions of covariates and (potential) outcomes, making the groups comparable in expectation (Shadish et al., 2002). The power of randomization will create what is known as a strongly ignorable assignment mechanism in Rubin's terminology (Imbens and Rubin, 2015; Rubin, 1974). Whenever the assignment mechanism is not enforced by the experimenter, but instead chosen by the individual confounding is a potential problem. For example, when individuals can self-select into a treatment, motivation can be an unobserved variable affecting both the assignment mechanism and the potential outcomes. In such a scenario the assignment mechanism is said to be confounded (Imbens and Rubin, 2015). The same is true for adherence or compliance in randomized trials because participants can choose whether or not to comply with treatment after it has been offered, and this opens up for unmeasured variables. Thus, the same analytical regression model (Eq. (3)) used to estimate ACE could be biased if we condition on an intermediate variable. Thus, randomization – or more generally an ignorable assignment mechanism – is the key and provides the basis for a fair comparison across treatment and control groups (Imbens and Rubin, 2015; Rubin, 1974).

In addition to an ignorable assignment mechanism (often accomplished through randomization of units), Rubin (Imbens and Rubin, 2015, pp. 11–13; Rubin, 2005) has specified the so-called *Stable Unit Treatment Value Assumption* (SUTVA) that, in turn, can be broken down into two aspects:

1. “No interference between units”: the potential outcome for any unit does not depend on the assignment of any other unit.
2. “No hidden variations of treatments”: for each unit, there is no different versions of treatment conditions that lead to other potential outcomes.

As one example of a violation of SUTVA, some participants in the control condition could get access to the text-based material of a guided internet-based psychological treatment via participants in the treatment condition and therefore receive a subpar version of the treatment without therapist guidance. Thus, there are now two forms of active treatments, one of which was not part of the manipulation (i.e., treatment without therapist guidance), and we would therefore have three, rather than two, potential outcomes. Another example would be a situation in which husband and wife are both included in the same treatment trial examining an individual-based psychological treatment. In such situation, their outcomes are likely to be influenced by their interaction (regardless of whether they are assigned the same or different conditions).

When SUTVA holds in randomized experiments, mean difference between treatment and control, $\hat{\beta_{1}}={\bar{y}}_{1}-{\bar{y}}_{2}$, is an unbiased estimate of *τ*. To include pre-treatment covariates that are related to the outcome will lead to a gain in precision (Imbens and Rubin, 2015). In a randomized trial it is also possible to get an unbiased estimate of *τ* conditional on such pre-treatment covariates (Imbens and Rubin, 2015). For example, we could be interested in the average effect of treatment for female participants only (i.e., ITT|female). However, as previously discussed, conditioning on an intermediate variable in similar fashion could bias the estimate. This is where principal stratification, and specifically, CACE-analysis comes in.

## 3 CACE-analysis: assumptions

CACE analysis is an extension of classic ITT-analysis. In CACE analysis, we aim to estimate the causal effect of assignment, that is the average difference in outcomes between treatment and control, but only within the subgroup of compliers (i.e., ITT|compliers). Similar to ITT, the causal effect at the individual level cannot be observed, but CACE can be estimated on the average under certain assumptions. For a continuous outcome, the population ACE for individuals in the subgroup of compliers is,

(4) $$\mathrm{CACE}=\tau_{c}=\mu_{1c}-\mu_{0c},$$

where *μ*_1*c*_ denotes the potential mean outcome for compliers when assigned to the treatment condition (*Z* = 1), and *μ*_0*c*_ denotes the potential mean outcome for compliers when assigned to the control condition (*Z* = 0). We can also view our overall ITT effect, *τ*, as the total effect consisting of the population average treatment effect among both noncompliers and compliers (Little and Yau, 1998). That is, *τ* = *π*_*c*_*τ*_*c*_ + (1 − *π*_*c*_)*τ*_*n*_, where the proportion for the compliers is *π*_*c*_, and the average treatment effect among noncompliers is *τ*_*n*_. If we solve for the treatment effect among compliers (i.e., CACE) we get,

(5) $$\tau_{c}=\frac{\tau -\left(1-\pi_{c}\right)\tau_{n}}{\pi_{c}}.$$

This seems simple enough, but the problem is that only one part of the information is observed in Eqs. (4), (5): we have access to the proportion of compliers and the outcome for the compliers in the treatment condition, but the same information is missing in the control. This is because those randomized to control never got access to treatment so we do not know how they would have responded under treatment. Given unobserved information on compliers membership and associated outcomes in control, if we wish to compare treatment and control for this subsample of participants we need to make additional assumptions. Angrist et al. (1996) have clarified the assumptions in the case of binary compliance, all or nothing compliance, using principal stratification. In principal stratification, individuals are classified on the basis of potential values of an intermediate variable, such as compliance status, under all treatment conditions (Frangakis and Rubin, 2002). The method allows researchers to estimate ACE within principal strata and this ensures that the variable, in this case compliance, does not influence treatment assignment and can therefore be treated as any other pre-treatment characteristics in a randomized trial (Frangakis and Rubin, 2002). In other words, a principal stratum is a homogeneous subgroup of individuals with regard to their potential outcomes.

In this particular case, we can now classify individuals into principal strata based on both binary assignment status (*Z*_*i*_) and binary compliance status (*T*_*i*_). That is, individuals can be classified according to whether they were assigned to treatment or not (*Z*_*i*_ = 1, treatment, *Z*_*i*_ = 0, control) and whether they complied with treatment or not (*T*_*i*_ = 1, complier, *T*_*i*_ = 0, noncomplier). Let then indicator *T*_*i*_ = *T*_*i*_(*Z*_*i*_) be the indicator of compliance status, where *T*_*i*_(1) is the compliance status for individual *i* when assigned to the treatment condition, and *T*_*i*_(0) is the compliance status for individual *i* when assigned to the control condition. Individuals can then be classified into four categories. Angrist et al. (1996) labeled these compliers, never-takers, always-takers, and defiers:

• *Compliers* are those that do what they are assigned to do, that is,
  $$T_{i}\left(1\right)=1\;\text{and}\;T_{i}\left(0\right)=0;$$
• *never-takers* do not take the treatment even when they are assigned to treatment, that is,
  $$T_{i}\left(1\right)=0\;\text{and}\;T_{i}\left(0\right)=0;$$
• *always-takers* take the treatment regardless of what they are assigned, that is,
  $$T_{i}\left(1\right)=1\;\text{and}\;T_{i}\left(0\right)=1;$$
• *defiers* do the opposite what they are assigned, that is,
  $$T_{i}\left(1\right)=0\;\text{and}\;T_{i}\left(0\right)=1.$$

In addition to randomization and SUTVA, there are two critical assumptions for CACE (Angrist et al., 1996):

1. Monotonicity: There are no defiers.
2. Exclusion restriction: There is no direct effect of treatment assignment among never-takers and always-takers. This also means that the outcomes among noncompliers are the same regardless of which conditions they have been randomized.

In trials where participants are prohibited by design from receiving a different condition than the one they were assigned, the number of noncompliance classes can be reduced to one: never-takers. In such situations, the monotonicity assumption also holds automatically because there are no defiers. In the current paper, I will focus on this form of noncompliance, so-called one-sided noncompliance (Imbens and Rubin, 2015). For simplicity, I will henceforth label the never-takers as noncompliers. The exclusion restriction assumption is still of importance and might be violated in trials evaluating psychological treatments where methods (e.g., blinding) to conceal assignment are not possible to use (Jo, 2002a). A commonly used example of a violation is when individuals who are randomized to treatment and who do not take the treatment are demoralized due to a missed opportunity. Such an effect is not observed under control because these individuals did not have access to treatment. In other words, there is a (psychological) effect of assignment among noncompliers (never-takers).

If the exclusion restriction assumption holds, the potential outcomes for noncompliers are the same regardless of assignment. In other word, there is no effect of treatment assignment among noncompliers, that is, *τ*_*n*_ = 0. In such situations CACE (Eq. (5)) equals,

(6) $$\tau_{c}=\frac{\tau }{\pi_{c}}.$$

Eq. (6) is known as the instrument variable estimator of CACE (Bloom, 1984; Imbens and Rubin, 1997; Little and Yau, 1998) and both quantities in Eq. (6) can be estimated using sample statistics (assuming that the denominator in Eq. (6) is above zero). The proportion of compliers in the population can be estimated, given that we have the sample proportions of compliers and noncompliers in the treatment condition, and on the basis of randomization, we can assume the same proportions in the control condition (Jo, 2002a). Thus, under these assumptions (SUTVA, Monotonicity, Exclusion restriction), CACE is identified in randomized experiment and can be estimated with observed data.

## 4 CACE estimation in the latent variable framework

Broadly categorized, there are two commonly used methods for estimating CACE: the instrument variable approach, which is simply the overall ITT effect divided by the proportion of compliers (Eq. (6)), and maximum-likelihood (ML) estimation approach (Jo, 2002a; Jo and Muthén, 2001; Little and Yau, 1998). I will focus on the latter, specifically, on a more recent approach to CACE estimation that is carried out within the framework of structural equation modeling using latent variables. I will do so for two main reasons. First, ML estimation is known to be more efficient than the IV approach (Imbens and Rubin, 1997; Little and Yau, 1998). Second, the latent variable approach using ML estimation is more flexible in terms of various extensions of CACE models (e.g., inclusion of covariates, growth models, multiple outcomes) (Jo and Muthén, 2001).

Within the latent variable framework for CACE estimation, compliance status is treated a finite mixture of subpopulations that can have different model parameters and distributions (Jo and Muthén, 2001). The principal strata of compliers membership all belong to a single latent class variable with proportions that sum up to 1, where the probability that an individual belongs to a certain class is to be estimated. Indeed, since the principal strata cannot be observed, it is natural to regard each stratum as belonging to a latent, unobserved, class variable (Jo and Muthén, 2001). In the cases of binary compliance, the latent class variable *C*_*i*_ contains two principal strata, or latent classes, compliers (*C*_*i*_ = *c*) and noncompliers (*C*_*i*_ = *n*). Compliers will recevie treatment, but only when they are assigned to treatment, whereas noncompliers will never receive treatment (i.e., never-takers) regardless of assignment. More formally,

(7) $$C_{i}=\left\{\begin{array}{ll} {\;}_{c}\left(\mathrm{complier}\right) & \;\text{if}\;T_{i}\left(1\right)=1\;\text{and}\;T_{i}\left(0\right)=0\\ {\;}_{n}\left(\mathrm{noncomplier}\right) & \;\text{if}\;T_{i}\left(1\right)=0\;\text{and}\;T_{i}\left(0\right)=0. \end{array}\right)$$

Compared to a conventional latent class analysis, or mixture model, where latent classes are unobserved subpopulations and classes are empirically derived (Lubke and Luningham, 2017; Muthén and Shedden, 1999), each class in CACE corresponds to a principal stratum that is a prior determined. That is, the classes are not affected by the treatment assignment and can be viewed as categories of an (unobserved) pre-treatment characteristics. In addition, the class variable is not completely unobserved; compliance class is observed in intervention but missing in the control. The incomplete information on compliance type can thus be viewed as a missing data problem (Little and Yau, 1998). The model uses the available information on class membership status from the intervention and the incomplete information on compliance status in control is handled as missing data using the EM algorithm. Similar to how missing data is handled in other situations of ML-EM estimation with incomplete data (see for an nontechnical explanation of ML-EM with missing data, Enders, 2010), all available information in the model (e.g., observed compliance information in the intervention as well as other observed auxiliary information in the model) is used to get ML estimates. For more technical details on ML-EM estimation in mixture modeling, and specifically in the context of CACE-analysis, the readers are referred to other sources (e.g., Jo and Muthén, 2001; Little and Yau, 1998; Muthén and Shedden, 1999).

Given that compliance type is unobserved in the control condition, only the overall mean *μ*_0_ for the control is observed. Thus, the distribution of the population mean for the control condition can be seen as a mixture of two unobserved distributions, compliers and noncompliers. (Jo et al., 2010; Jo and Muthén, 2001). In the binary compliance case, the mixture is, *μ*_0_ = (1 − *π*_*c*_)*μ*_0*n*_ + *π*_*c*_*μ*_0*c*_, where the proportion for the compliers is *π*_*c*_, and the potential outcome mean for noncompliers in the population is *μ*_0*n*_. Solving for the population mean of compliers in the control *μ*_0*c*_ yields, *μ*_0*c*_ = (*μ*_0_ − (1 − *π*_*c*_)*μ*_0*n*_)/*π*_*c*_. Under the exclusion restriction assumption that noncompliers are not affected by assignment and should therefore have same population means in treatment and control (i.e., *μ*_1*n*_ = *μ*_0*n*_), CACE (Eq. (4)) can now be rewritten as,

(8) $$\tau_{c}=\mu_{1c}-\left\{\frac{\mu_{0}-\left(1-\pi_{c}\right)\mu_{1n}}{\pi_{c}}\right\}.$$

All parameters in Eq. (8) are directly estimable from the observed data and can be estimated using ML (Jo et al., 2010).

CACE for a single continuous outcome *Y* for individual *i* within latent class *k* can then be evaluated using the following linear model (Jo and Muthén, 2001),

(9) $$Y_{\mathrm{ik}}=\alpha_{k}+\gamma_{\mathrm{Zk}}Z_{i}+\epsilon_{\mathrm{ik}},$$

where *α*_*k*_ is the mean for the control group within compliance class *k*, and *γ*_*Zk*_ is the treatment effect within class *k*. The residual term *ϵ*_*ik*_ within each class *k* has a variance *σ*_*k*_^2^ that is normally distributed with a mean of zero. The class variable *C* contains two levels (*K*), or classes (*k* = 1, for compliers, *k* = 2, for noncompliers), where the proportion for the compliers is, *π*_1_, and the proportion for noncompliers is, 1 − *π*_1_ = *π*_2_. As previously discussed, compliance is observed in the treatment condition but missing in the control. Just as ordinary regression we can obtain the mean for the control and treatment within each compliers class. The means for control and treatment among noncompliers are, *μ*_0, *k*=2_ = *α*_2_ and *μ*_1, *k*=2_ = *α*_2_ + *γ*_*Z*2_, respectively. Similar, the means for control and treatment among compliers are *μ*_0, *k*=1_ = *α*_1_ and *μ*_1, *k*=1_ = *α*_1_ + *γ*_*Z*1_, respectively.

As we only have the overall mean in the control, note that the two means for compliers and noncompliers, *α*_1_ *α*_2_, in control group are unobserved. Thus, we need the exclusion restriction assumption for identification. As per the exclusion restriction assumption, the treatment assignment effect among noncompliers (*k* = 2) is constrained to zero *γ*_*Z*2_ = 0, making the mean among noncompliers in control equal to the mean of noncompliers in treatment, that is, *α*_2_ = *μ*_1, *k*=2_. CACE (Eq. (8)) can now be re-expressed in terms of known quantities as,

(10) $$\tau_{c}=\gamma_{Z1}=\mu_{1,k=1}-\left\{\begin{array}{l} \frac{\mu_{0}-\left(1-\pi_{1}\right)\mu_{1,k=2}}{\pi_{1}} \end{array}\right\}.$$

The model (Eq. (9)) can easily be extended by including covariates that predict outcome. A natural extension is to include the baseline value of the outcome as a covariate in the model, making it to an ANCOVA model. Any other pre-assignment covariate that is related to the outcome could also be included to increase precision. The effects of the covariates can be constrained to be the same across both classes or we can relax this assumption and allow effects of covariates to vary across classes. The CACE model with a covariate *X*_*i*_ is,

(11) $$Y_{\mathrm{ik}}=\alpha_{k}+\gamma_{\mathrm{Zk}}Z_{i}+\gamma_{\mathrm{Xk}}X_{i}+\epsilon_{\mathrm{ik}},$$

where *γ*_*Xk*_ represents the effect of the covariate within class *k*.

In addition, we can also include covariates to predict compliance status. This is of substantive interest as we might want to know which participants are likely to be compliers in a study. In addition, this can increase power in CACE and decrease bias due to model misspecifications (Jo, 2002a; Stuart and Jo, 2015). With only two classes, the model is a logistic regression model examining the probability of being a complier (*π*_1*i*_) as a function of a set of covariates *x*_*i*_, *logit*(*π*_1*i*_) = *β*_0_ + *β*_*i*_*x*_*i*_, where *β*_0_ is a logit intercept and *β*_*i*_ a vector of logit coefficients (Jo, 2002a; Jo and Muthén, 2001).

**Fig. 1:**
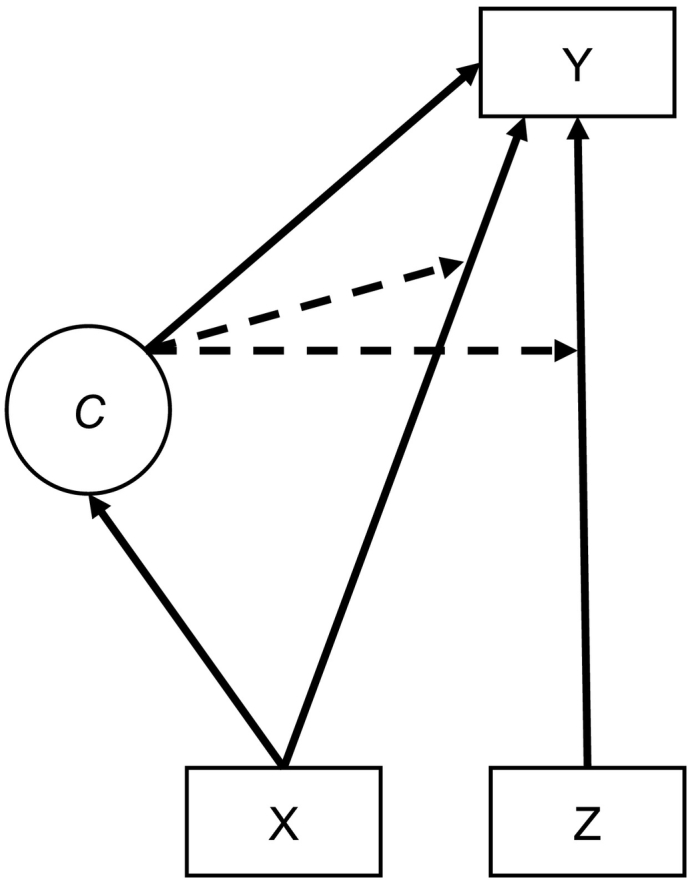
CACE analysis of a continuous outcome (Y) regressed on a binary treatment indicator (Z) and an additional covariate (X).

Although the model may seem complicated, it can be readily implemented in a structural equation program (e.g., Mplus) and everything is estimated jointly using ML estimation. Fig. 1 depicts the model with a covariate (X) predicting both outcome (Y) and compliance status (*C*). In the figure, squares represent observed variables and the circle is the latent class variable containing information about compliance status. Solid lines from observed variables correspond to regressions among variables. Specifically, the path from Z (binary treatment variable) to Y (outcome) represents the treatment effect. The dotted lines that originate from the latent class variable (*C*) to the regression of the observed variables (Z, X) on the outcome signify that the effects on the outcome are allowed to vary across compliance class. The solid line from the latent class variable to the outcome indicate that the means for the noncompliers and compliers are allowed to be different.

The model can be estimated in Mplus and the input file for the model in Fig. 1 is provided in Appendix 1 (for more details on Mplus syntax see the user manual, Muthén and Muthén, 2017). To be able to specify that the classes are known in intervention but missing in control, the TRAINING option in Mplus is used together with two observed variables (c1, c2) containing information on complier class membership. Individuals in the treatment are assigned values of 1 for c1 and 0 for c2 if they are noncompliers and 0 for c1 and 1 for c2 if they are compliers. Individuals in the control group are assigned values of 1 for both c1 and c2 to indicate that that their class membership is unknown. The CLASSES command specifies the name of the latent class variable, c, with the number of classes to be used in parenthesis (i.e., 2) with mixture modeling, that is, TYPE = MIXTURE. The model estimated in both classes are provided under the %OVERALL%. Here, the outcome y is regressed on the binary treatment variable (z) and an additional covariate (x). The compliance status (c) is also regressed on the covariate. The class-specific effects are presented under c#1 and c#2. The effect of treatment is fixed to zero in the noncompliers class (c#1), whereas it is freely estimated in the compliers class (c#2). The means (intercepts) of the outcome, the effect of the covariate on the outcome and the residual variance are all freely estimated in each class, that is to say, they are allowed to vary across class. The effect of the covariate and residual variance can be constrained to be equal across classes by removing the class-specific statements in the input file.

## 5 CACE-analysis: illustrative example

To demonstrate CACE estimation, data from a previously published guided internet-based psychological treatment for irritable bowel syndrome (IBS) (Ljótsson et al., 2014) will be used. In this study, individuals (*N* = 309) were randomly assigned to treatment and an active control condition. The study was designed to isolate a core component (exposure therapy to IBS symptoms and situations) of a previously well-validated psychological treatment for IBS (Ljótsson et al., 2011). The only difference between the two conditions was that in the treatment condition individuals received the full treatment package, whereas in the control one of the core treatment components was removed. We could record whether any individual in the intervention received access to the treatment module of primary interest in the trial. Thus, in the study a complier was defined as a person who had gained access to the treatment module containing the text describing the treatment component that was isolated in the trial. Using this definition, 55% of individuals were classified as compliers in the treatment condition. As individuals randomly assigned to control were not given access to the isolated treatment component, never-takers were the only type of noncompliance that was possible in the trial. Previously reported ITT-analysis showed that the effect favored the treatment at post-treatment assessment with a small-to-moderate effect size, but a re-analysis using CACE-analysis demonstrated that this effect was substantially underestimated (Hesser et al., 2017).

The analytical model used here to demonstrate CACE is the regression model (as in Eq. (11); depicted in Fig. 1), where the post-outcome measure of IBS-symptoms was regressed on a binary-treatment variable and the pre-treatment values of the same outcome measure (similar to ANCOVA). To be able to compare ITT, per-protocol and CACE-analysis, a model implied unstandardized mean difference and an effect size (*d*) at endpoint were computed for each model. The effect size was the mean difference between conditions divided by the estimated standard deviation (from the ITT-analysis). The ITT analysis provides an overall causal effect of assignment, but ignores the fact that almost half of the participants did not receive the treatment component that was actually tested in the trial. As such, it may have underestimated the effect of treatment. The per-protocol analysis used the same model as the ITT, but included only individuals who were classified as compliers in the treatment and compared results with all individuals in the control (*N* = 240). The estimate is not causal in the same sense as ITT or CACE, but the analysis is performed to be able to contrast the results from the other models. Finally, the CACE analysis used the same overall model as the ITT, but this model also took into account compliance status by incorporating a latent class variable in the regression model. The effect of assignment among noncompliers (never-takers) was constrained to zero (as per the exclusion restriction assumption), whereas the effect among compliers was freely estimated. To make CACE comparable to the other models, the effect of the covariate on outcomes was constrained to be the same in both classes. All models were estimated within the structural equation modeling framework using Mplus (Muthén and Muthén, 2017) and full information maximum likelihood estimation with non-normality robust standard errors.^1^ Table 1 shows the primary results of these models.

**Table 1:** Treatment effects from estimated models.

Analysis	Estimate	Standard error	*z*-Value	*p*-Value	Effect size
ITT	−4.09	1.145	−3.571	<0.01	0.417
Per-protocol	−5.651	1.271	−4.446	<0.01	0.577
CACE	−7.483	2.293	−3.264	<0.01	0.764

Note. CACE = complier average causal effect; ITT = intention-to-treat.

As can be shown in Table 1, CACE analysis was almost twice the size of ITT (effect size 0.76 vs. 0.42) and also substantially higher than per-protocol (effect size = 0.58). These estimates were similar to those reported in the earlier publications, but differed slightly given analytical models (ANCOVA vs growth models) and covariates in the model (see Hesser et al., 2017). More importantly, the difference in results between CACE and ITT demonstrate the importance of performing CACE analysis. In other words, different conclusions in terms of effects were reached depending on the analytical model, CACE or ITT.

Additional covariates can be included in CACE-analysis to examine possible associations with outcome and compliance type. As stated earlier, the motivation for inclusion of covariates in the model are multiple. First, by regressing the outcome of a set of baseline covariates that are strongly related to outcome there is a gain in precision. Second, this allows us to examine separate associations between baseline characteristics and outcome within the class of compliers and noncompliers. Third, by regression compliance class membership on a set of covariates statistical power can be increased and bias due to model misspecifications can be reduced. Fourth, this logistic regression on compliance types allows us to examine which characteristics that are associated with being a complier.

To demonstrate how this is accomplished, I re-estimated the CACE model with an additional set of covariates; for demonstration purposes only with four demographic variables (employment, female, age, university education). These covariates were allowed to influence both outcome and compliance status and the effects of the covariates on outcome were allowed to vary across compliers and noncompliers. The results are presented in Table 2. As can be observed, only two predictors were statistically significant among compliers. University education and age were both negatively associated with the outcome, suggesting that having a university education and higher age were associated with better treatment outcome (i.e., lower IBS-symptoms), but only within the subgroup of compliers. None of the covariates in logistic regression could predict the probability of being a complier. The estimate of CACE was similar to the one from the previous model that only included the pre-treatment value of the outcome as the covariate.

**Table 2:** Results from CACE-analysis with covariates.

Predictor	Estimate	Standard error	*z*-value	*p*-value
Regression on outcome				
Noncomplier				
Z	0^a^			
Employment	2.397	2.166	1.106	0.269
University education	2.215	2.751	0.805	0.421
Age	−0.003	0.069	−0.048	0.962
Female	−1.784	2.072	−0.861	0.389
Complier				
Z	−7.581	2.499	−3.034	0.002
Employment	−3.071	1.96	−1.567	0.117
University education	−5.877	2.466	−2.383	0.017
Age	−0.131	0.066	−2.007	0.045
Female	−0.951	2.214	−0.43	0.667

Regression on class (complier vs. noncomplier)				
Employment	0.131	0.369	0.354	0.723
University education	−0.567	0.397	−1.429	0.153
Age	−0.002	0.013	−0.143	0.886
Female	−0.322	0.379	−0.851	0.395
Effect size, CACE	0.774			

a Constrained to zero as per the exclusion restriction assumption. Z is the binary treatment variable.

## 6 Model extensions and analytical considerations

Although it is beyond the scope of this paper to provide the specific details, there are a number of possible model extensions given a high degree of flexibility when estimating CACE within the latent variable framework. Analytical models can accommodate multiple outcomes in the same model, non-continuous outcomes (e.g., categorical outcomes), and outcomes measured repeatedly over time (Jo and Muthén, 2001). For example, the re-analysis of the IBS-trial employed a growth models as the primary analytical model for CACE estimation (Hesser et al., 2017). Thus, instead of having to rely on a single endpoint value, repeated assessments of the outcome during the treatment phase allowed us to compare growth trajectories across conditions during qualitative distinct phases of the trial, prior to and following implementation of the specific treatment module that was examined in the IBS-trial. In addition to providing greater details on the effects of treatment, such auxiliary information (e.g., growth trajectories) can also increase the precision of CACE estimates (Jo and Muthén, 2003). In fact, whenever more than two measures are available the use of growth models is a natural extension of CACE-analysis. This is readily accomplished in the latent variable framework by including continuous latent variables, random effects that stipulate person-specific trajectories of change (Hesser, 2015; Muthén and Curran, 1997), in addition to the categorical latent class variable (i.e., this combination of continuous and categorical latent variables is often referred to as growth mixture models).

By using covariates in the model, the exclusion restriction assumption can also be relaxed and tested (Jo, 2002b). As stated earlier, the exclusion restriction is a key assumption for model identification, but could be violated in trials of psychological treatments. Sensitivity analysis is often recommended and this is readily done within the structural equation modeling framework by allowing treatment assignment to have an nonzero effect in the noncompliers class, under other identifications than the exclusion restriction assumption (for more information on alternative identifications of CACE see, Jo, 2002b).

An interrelated aspect of adherence is missing data. The models used here for demonstration of CACE-analysis assumed that missing data was ignorable conditioning on the other observed variables in the model (i.e., missing at random [MAR]; Enders, 2010). The MAR assumption is a fairly reasonable one and maximum likelihood estimation is often recommended method for handling missing data in clinical trials (Enders, 2010; Hesser, 2015; Salim et al., 2008). In fact, this is another advantage of using CACE within a structural equation modeling framework as ML-EM estimation is the default in most structural equation modeling software (e.g., Mplus). In addition, where researchers suspect that the MAR assumption is not tenable, alternative model specifications for CACE, with non-ignorable missing data assumptions, have been developed and implemented in the latent variable framework (Jo et al., 2010). It should be noted that in such scenarios the ITT-effect is also biased as the analysis need to take into account principal strata based on both compliance and missing data (Frangakis and Rubin, 1999).

Finally, in this demonstration of CACE I focused on binary compliance status, all-or nothing compliance. In some cases we may suspect varying degree of compliance and models have also been developed for such situations (Jin and Rubin, 2008), but, as far as I am aware, they have not been adopted to trials evaluating psychological treatments. In view of this, one alternative is to use and test multiple definitions of adherers based on different dosages of treatment exposure and compare results. Mixture modeling, latent class analysis, could also be used, in a conventional way, as an exploratory device to identify latent discrete classes based on observed adherence behavior in treatment in a first step, and these empirically derived groups can then form groups in CACE-analysis in a second step (see, Jo and Muthén, 2003). In addition, methods have also been developed that focus on further dividing the subgroup of compliers into more than one effect-class (Sobel and Muthén, 2012).

## 7 Final recommendations

The objective of the paper was to promote an analytical method that permits causal inference in randomized trials in the context of a very common problem in internet interventions, nonadherence. The paper was written as a gentle introduction to applied researchers to encourage the use of CACE-analysis in the field. CACE-analysis is an analytical method for researchers to answer substantive research questions. Most importantly, it provides an estimate of the effect of the treatment itself among a subsample of participants who take the treatment when it is offered (and do not take it otherwise) and may therefore provide information on the effect of the treatment itself or treatment recipient, not just the effect of offering treatment or assignment of treatment (Imbens and Rubin, 2015; Sobel and Muthén, 2012; Stuart et al., 2008). It should be noted that I am not arguing against performing ITT-analysis in randomized trials. Rather, both analytical models have important, albeit different, roles to play. In fact, while the ITT-effect remains agnostic about postassignment variables such as adherence, it would still constitute the primary estimate in an RCT given that modern sophisticated methods, such as CACE, require considerably stronger assumptions than ITT (Sagarin et al., 2014).

A number of aspects should be taken into account before using CACE as a complimentary tool. First, as usual with any analytical method, careful consideration should be given to the plausibility of the underlying assumptions, since they cannot be empirically verified. This also applies to ITT-analysis (and causal inference more generally), but CACE has additional assumptions that may not be plausible. In many situations that involve complex trials, such as psychological interventions, plausibility of model assumptions are often controversial (see for a discussion regarding SUTVA in psychological treatments, Shadish, 2010). As previously stated, the exclusion restriction assumption can be violated and subsequently bias the estimate. Violations can result from an imperfect measure of compliance, partial compliance or psychological effects of assignment. The assumption is potentially especially problematic for non-medical trials, where blinding is often not feasible (Imbens and Rubin, 2015; Little et al., 2009). When one suspects violations, sensitivity analyses to assess the extent to which violations might bias the estimate are warranted. Indeed, sensitivity analyses are commonly regarded as essential component of causal inference with intermediate variables more generally as such inference is always based on unverified assumptions (Stuart and Jo, 2015; VanderWeele, 2015). In many situations, it may be necessary to compare results with different identification strategies and model assumptions to arrive at valid conclusions and methods have been developed that can provide informative bounds of causal effects by jointly considering alternative sets of identifying assumptions (Jo and Vinokur, 2011).

In this context, it should also be noted that CACE-analysis is not the only approach to handle nonadherence in randomized experiments and other methods may be more appropriate in certain circumstances (see for review, Sagarin et al., 2014). For instance, it was recently argued that the instrument variables methods, such as CACE, may be a viable alternative when examining a single intervention at single time point, but may not be appropriate in more complex treatment studies which require sustained adherence over time (Hernán and Robins, 2017). In the latter case, statistical adjustment for observed variables using other statistical techniques (e.g., G-estimation methods) could be an option. On the other hand, the validity of such methods requires data on all preassignment and postassignment prognostic factors predictive of adherence which may not be available in a trial and these “modern” per-protocol analyses have not systematically been compared with classic instrument variables methods (Hernán and Robins, 2017). Which approach that should be taken will depend on the questions of substantive interest and which assumptions that are more plausible in a specific setting. This in turn relies heavily on subject-matter knowledge, including treatment theory and design-aspects. In general, CACE appears most useful in settings where one well-defined treatment is compared with a control at specific time point, compliance is binary and careful restriction of treatment access is guaranteed, but CACE has also been used in more complex situations (e.g., Jo and Muthén, 2003; Little et al., 2009; Stuart et al., 2008).

Moreover, and more importantly, it is crucial to consider the broader context in which any analytical data choice is made. In order words, researchers need to consider design aspects that can reduce threats to internal and external validity. For instance, the type of control condition used in an RCT will determine what kind of alternative explanations that are ruled out (Shadish et al., 2002). Indeed, within the potential outcome framework, the causal effect is always relative to the outcome under control, as the effect is defined as the difference between the two causes (Holland, 1986). Thus, the framework offers a way to solve a causal identification problem that involves unobserved counterfactuals, but researcher still need to think carefully about what kind of a “causal question” their study can answer and the specific counterfactual under consideration. In this context, it should also be noted that CACE-analysis does normally not answers questions about how or why change occur in treatments (i.e., mediation), although principal stratification has been adopted to statistical mediation analysis (Jo, 2008). VanderWeele, 2012, VanderWeele, 2015 provided an in-depth discussion on the differences between principal stratification applied to noncompliance and mediation analysis in the form of natural direct and indirect effects. It also important to remember that we normally test for average causal effects in the context of considerable individual heterogeneity and average effects may not be easily – or at all – extrapolated to the individual. At the end of the day, no single study can provide definitive proof of a causal association nor the degree to which it can be generalized. Rather, the investigation of causal effects requires a multifaceted pattern of evidence obtained from multiple studies (Shadish et al., 2002). Viewed in this light, any data analytical approach can only make a small contribution to a complex scientific task. Still, given the amount of effort and time involved in conducting an RCT, researcher should be advised to extract as much relevant information as possible and appropriate analyses that take into account adherence may be informative. At the same time, researchers should be aware of these methods limitations.

Notwithstanding these important specific and general considerations, CACE, carried out within the latent variable framework, is probably one of the most flexible approaches, and if used correctly can provide valuable complementary information in certain settings. Despite its potential value, CACE-analysis is underutilized in trials on psychological treatments. One reason could be that it is difficult to define what constitutes an adherer in a psychological treatment. Indeed, an accurate measure of adherence is a prerequisite for CACE to be of value. This is potentially the most challenging aspect of CACE-analysis. I recognize that this is not an easy thing to do in any trial, but, as discussed earlier, it is often trickier in face-to-face treatments to determine whether a participant has been exposed to the treatment content, because it relies on that we know what therapists are doing in treatment. In internet interventions we often have more control over the content of the treatment and what participants are exposed to as the therapist often only have a supportive role (or no role at all in pure self-help treatments). We should therefore be able to determine more easily whether or not a participant has received treatment as intended.

It should be noted that the definition of adherence used in the current paper is based on what participants receive in treatment or what content they are exposed to, not on what they actually do in treatment, in terms of, for example, exercises and assignments. Participants may, for example, log in and read the treatment text in an internet-based psychological treatment, but this does not automatically mean that they are also engaged in treatment (see e.g., Bendelin et al., 2011). The degree to which participants are engaged in treatment is of course another important aspect of adherence in internet interventions (Donkin et al., 2013; Kelders et al., 2012), although arguably a more challenging one from a methodological standpoint. Here, the format of internet-based treatment may also have an advantage over traditional means of delivering treatment given the many objective measures that are regularly collected in internet interventions, such as number of log ins, time spent online, number of completed activities etc. (Donkin et al., 2013). With more advanced assessment technology (e.g., ecological momentary assessment) being incorporated into internet interventions we may also be able to get a more fine-grained picture of this kind of adherence. Such information, coupled with for example data-intensive methods such as machine learning (e.g., Mikus et al., 2018), could be used to identify adherers. In this context, it should be noted that CACE-analysis is, of course, not limited to the current conceptualization of adherence and various sources of information could be used to classify participants. There is often no single, best way to determine adherence in a treatment trial and, in most situations, researchers should entertain multiple definitions and compare results across models with different definitions. CACE-analysis, within the latent variable framework, provides the analytical tools to do just that.

Let me end with a final set of recommendations when dealing with nonadherence in internet interventions:

• Per-protocol and as-treated analysis are not recommended as the methods do not provide causal estimands. The same applies to simpler but commonly used methods examining dose-response effects in internet interventions, such as, for example, estimating the correlation between the number of completed modules with the outcome or conditioning on any other post-assignment variable measuring the degree of exposure to treatment.
• Researchers should, whenever possible, a prior define an adherer in a particular intervention. Measures of adherence should be collected in the trial so that participants can be classified according to this predetermined definition. Whenever continuous measures of adherence are dichotomized, sensitivity analyses can be carried out using different thresholds to see how choices of cut-off impact the findings.
• CACE-analysis, whenever it is considered appropriate, should be carried out as complement to ITT-analysis in randomized trials with a control condition. Sensitivity analysis of the exclusion restriction assumption should be performed to test whether any violations alter the overall conclusions. Pre-treatment characteristics that are related to the outcome or compliance should be included in the model to increase precision and power and decrease bias due to model misspecifications.

The following is the supplementary data related to this article.

Appendix 1 Mplus syntax for CACE analysis. Appendix 1

## Declaration of competing interest

The author declares that he has no known competing financial interests or personal relationships that could have appeared to influence the work reported in this paper.

## Footnotes

1 There was a small amount of missing data at post-assessment across conditions (*n* = 17). To be able to incorporate all individuals in the models, the variance of the pre-treatment value of the outcome was brought into the model. Mplus treats this variable as any other dependent variable in the model and thus returns ML estimates with incomplete data under the missing at random assumption and multivariate normality (Muthén et al., 2016).
