# Supplementary material for: Estimating causal effects of internet interventions in the context of nonadherence
Source: Internet Interv. 2020 Aug 29;21:100346. doi: 10.1016/j.invent.2020.100346 (PMC7495102; doi:10.1016/j.invent.2020.100346)
Supplement: Appendix 1 — Mplus syntax for CACE analysis. [file mmc1.docx]

**APPENDIX 1.**

**Mplus syntax for CACE analysis**

Note: anything on a line to the right of **!** is ignored by Mplus.

| TITLE: CACE estimation with one additional covariate |
| --- |
| DATA: FILE IS CACE.dat; |
| VARIABLE: NAMES ARE **y z x c1 c2;**  **!outcome, y, binary treatment indicator, z, covariate, x.**  **!binary indicators, c1 c2, containing information on compliance class membership (see !article).** |
| **CLASSES = c (2);**  **!latent categorical variable, c, with two classes, two principal strata.** |
| **TRAINING = c1 c2;**  **!variables that are used together with mixture modeling with information on principal !strata, compliance class membership** |
| USEVARIABLES ARE y z x c1 c2;  ANALYSIS: **TYPE = MIXTURE;** |
| **!fitting a mixture model (a latent class model)**  MODEL: |
| **%OVERALL%** |
| **y ON z x;** |
| **c ON x;**  **!These command lines specify the models that apply to both classes. Here, the outcome y**  **!is regressed on the binary treatment indicator and the covariate. The second line is the !logistic regression model for compliance membership** |
| **%c#1%** **!noncompliers** |
| **[y]; y;**  **!mean and variance, respectively, freely estimated in noncompliers class (i.e. not !constrained to be equal across compliers and noncompliers)** |
| **y ON z@0;**  **!effect of treatment assignment constrained to zero in the noncompliers class as per the !exclusion restriction assumption** |
| **y on x;**  **!the effect of covariate in the noncompliers class freely estimated (i.e. not constrained to !be equal across compliers and noncompliers)** |
| **%c#2% !compliers** |
| [y]; y; |
| **y ON z;**  **!the freely estimated effect of treatment assignment in the compliers class** |
| y on x; |
|  |
